# Correction: Cu-catalyzed C–C bond formation of vinylidene cyclopropanes with carbon nucleophiles

**DOI:** 10.1039/c9sc90262g

**Published:** 2020-01-10

**Authors:** Jichao Chen, Shang Gao, Ming Chen

**Affiliations:** Department of Chemistry and Biochemistry, Auburn University Auburn AL 36849 USA mzc0102@auburn.edu

## Abstract

Correction for ‘Cu-catalyzed C–C bond formation of vinylidene cyclopropanes with carbon nucleophiles’ by Jichao Chen *et al.*, *Chem. Sci.*, 2019, **10**, 10601–10606.

We regret that in the original article the structure of compound 1 in Tables 1–3 was incorrect. The correct structure is given below.
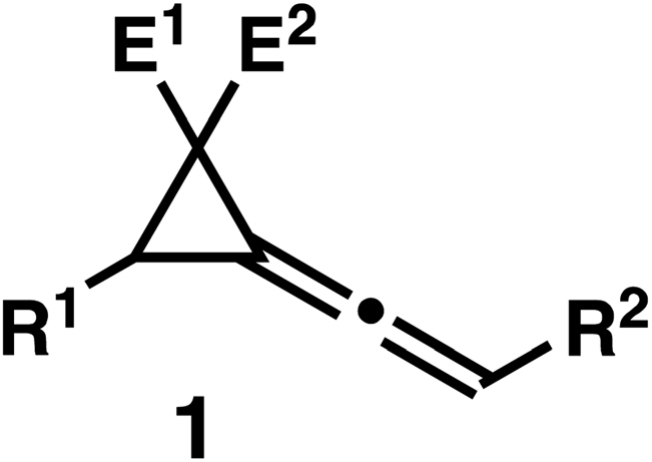


The Royal Society of Chemistry apologises for these errors and any consequent inconvenience to authors and readers.

## Supplementary Material

